# Methods in belowground botany

**DOI:** 10.1002/aps3.1239

**Published:** 2019-04-09

**Authors:** Gregory J. Pec, Megan K. Ljubotina, James F. Cahill

**Affiliations:** ^1^ Department of Natural Resources and the Environment University of New Hampshire Durham New Hampshire 03824 USA; ^2^ Department of Biological Sciences University of Alberta Edmonton Alberta T6G 2E9 Canada

The study of plant root systems is not new (Darwin and Darwin, 1880), but it has received increased attention in recent decades with technological advancements in molecular genetics (Cooper and Rao, 2006) and efforts to standardize procedures (e.g., root sampling methods based on anatomy, morphology, and physiology) in field ecology (Freschet and Roumet, 2017). Plant root systems play an integral role in numerous ecological processes, for example, from building soil structure (Rillig et al., 2015), to impacting species composition (Craine and Dybzinski, 2013), as well as mediating carbon and nutrient cycling within ecosystems (Bardgett et al., 2014). Specifically, roots account for up to 63% of the plant's total biomass (Poorter et al., 2012) and up to 60% of net primary productivity in many ecosystems (McCormack et al., 2015). Despite their ecological importance, our understanding of plant root systems and their interactions with other plants, microbes, and the environment is often limited due to methodological challenges associated with their hidden nature in the soil matrix. In this special issue of *Applications in Plant Sciences*, “Methods in Belowground Botany,” we present six papers that explore current methods and challenges in investigating plant root systems, ranging from the subcellular to the ecosystem level, with a wide variety of applications that advance our understanding of belowground botany.

## SUBCELLULAR LEVEL

Our first paper in this issue focuses on a rapid and useful technique for evaluating the capture of environmental lead by maize root cap border cells. Lead is a toxic metal that is released into the environment largely due to mining, agriculture, and other industrial activities (Holmgren et al., 1993; Baran et al., 2014). Recent concerns regarding the contamination of soils by toxic metals such as lead has sparked efforts toward their removal. Here, Huskey et al. (2019) use rhodizonic acid as an indicator to detect the trapping of lead by the border cells of corn root tips. The authors use the method to demonstrate the potential importance of extracellular DNA in trapping lead and protecting the plant by comparing the dynamics of the interaction in the presence and absence of nucleases. Potential applications in phytoremediation are discussed.

## PLANT LEVEL

Improvement of crop rooting systems has been identified as a key component in the development of more stress‐tolerant and agronomically important crop plants (Paez‐Garcia et al., 2015), and ultimately in achieving edible yields to address the challenge of sustaining an ever‐growing human population. In Kengkanna et al. (2019), the authors characterize phenotypic variation in cassava root traits using a high‐throughput method for the first time in a perennial root crop. The authors find that this high‐throughput root phenotyping method using the software digital imaging of root traits (DIRT) yields results that are highly correlated with manual measures of root traits. They also demonstrate variation in root traits among different genotypes and suggest that such high‐throughput methods could allow for selective breeding for root traits that allow cassava to better adapt to drought.

Along with the difficulties of characterizing phenotypic variation in roots and determining the relative importance of certain root traits, the identification of roots to species in mixed samples has long been a challenge in belowground botany (Rewald et al., 2012). Fluorescent amplified fragment length polymorphisms (FAFLPs) are a low‐cost, high‐throughput method for identifying multiple species in a single sample. Here, Metzler et al. (2019) test the efficacy of FAFLPs for identifying mixed samples of roots and provide new size profiles for 193 species, effectively doubling the previous number of available reference species. They find that the FAFLP method can be as effective or more effective in detecting species than traditional Sanger sequencing, although ambiguous species identifications in mock communities increase with species richness.

The last paper in this section provides insight into integrating historical data with the application of molecular techniques to monitor the effects of global changes over time. Heberling and Burke (2019) use the roots of herbarium specimens to quantify historical arbuscular mycorrhizal fungal communities by amplifying arbuscular mycorrhizal fungi (AMF) DNA. They demonstrate that this method can be used to detect differences among plant species in AMF community composition. They suggest that herbarium curatorial practices may be adjusted to better maximize this potential use. Surprisingly, AMF DNA on the oldest specimens was amplified successfully at a higher rate than on newer specimens, perhaps because modern herbarium practices such as drying specimens at higher temperatures can degrade DNA.

## COMMUNITY LEVEL

Litter decomposition is a key component in maintaining carbon and nutrient cycling within ecosystems and is influenced by a suite of abiotic and biotic factors, including, in particular, soil microorganisms (Hobara et al., 2014). Martini et al. (2019) outline a methodology for assessing differences in litter decomposition on a fine scale in a high‐diversity forest. In doing so, they test the home‐field advantage hypothesis that microbial communities under trees of a given species will be better adapted to decompose the litter of that species. They also demonstrate fine‐scale variation in microbial biomass and soil nutrients, as well as in correlations between soil nutrients among parts of the forest floor under different tree species. These findings highlight the importance of belowground methodologies that can account for fine spatial differences, especially in highly diverse plant communities.

## ECOSYSTEM LEVEL

The remaining contribution to this issue highlights techniques that can be used with data logger technology to obtain valuable information, such as microhabitat variation in temperature and soil moisture, and demonstrates the importance of fine‐scale spatial variability in understanding belowground plant ecology. Fawcett et al. (2019) highlight three case studies where the use of iButtons allowed the authors (1) to characterize differences between modeled climate data and microhabitat‐level data for a rare fern, (2) to characterize fine‐scale variation in temperature for predicting phenology of wild chickpea, and (3) to characterize changes in microhabitat caused by a solar array installation. Additionally, authors provide MATLAB code to facilitate the processing of raw iButton data.

Overall, the papers in this issue present an overview of work at the cutting edge of belowground botanical research. Molecular genetic approaches coupled with consistent field methods are becoming more standard in elucidating relationships between plant root systems, microbes, and their environment. As the application of these technologies and techniques continues to develop, we will gain the ability to extend beyond belowground species identification and delve into patterns of nutrient and trophic dynamics with increasingly rapid and robust methods. The future of belowground research looks bright—in this special issue, we hope that you will find both information about the current state and inspiration for forthcoming work in this dynamic area of plant sciences.
